# Effects of deep cervical flexor training on impaired physiological functions associated with chronic neck pain: a systematic review

**DOI:** 10.1186/s12891-018-2324-z

**Published:** 2018-11-28

**Authors:** Johannes Blomgren, Erika Strandell, Gwendolen Jull, Irene Vikman, Ulrik Röijezon

**Affiliations:** 1 Department of Health Sciences, Luleå University of Technology, Luleå, Sweden; 2 Physiotherapy, School of Health and Rehabilitation Sciences, The University of Queensland, St Lucia, Brisbane Australia

**Keywords:** Neck pain, Deep cervical flexor training, Strength training, Physiological outcome measures, Systematic review

## Abstract

**Background:**

Neck pain is a major health issue with high rates of recurrence. It presents with a variety of altered sensorimotor functions. Exercise is a cornerstone of rehabilitation and many training methods are used. Exercise is evaluated in most randomized controlled trials on its pain relieving effects. No review has assessed the effect of exercise on the altered physiological functions or determined if there are differential effects of particular training methods. This review investigated the effects of deep cervical flexor (DCF) training, a training method commonly used for patients with neck pain, and compared it to other training methods or no training on outcomes of cervical neuromuscular function, muscle size, kinematics and kinetics.

**Methods:**

Web of Science, Scopus, CINAHL, PubMed were searched from inception until January 2018. Twelve randomized controlled trials were included that compared DCF training as sole intervention to other training or no interventions in persons with neck pain. The Cochrane Risk of Bias tool was used to assess the method quality. All outcome measures were analysed descriptively and meta-analyses were performed for measures evaluated in three or more studies.

**Results:**

DCF training was compared to cervical endurance, strength, proprioception and mobility training, muscle stretching, and no intervention control groups. Physiological outcome measures included neuromuscular co-ordination (craniocervical flexion test), functional tasks, muscle fatigability, muscle size, kinematics (joint position sense, posture and range of motion) and kinetics (strength, endurance and contraction accuracy). Strong evidence was found for effectiveness of DCF training on neuromuscular coordination, but it had no or small effects on strength and endurance at higher loads. DCF training improved head and cervical posture, while evidence was limited or contradictory for other measures.

**Conclusions:**

DCF training can successfully address impaired neuromuscular coordination, but not cervical flexor strength and endurance at higher contraction intensities. A multimodal training regime is proposed when the aim is to specifically address various impaired physiological functions associated with neck pain.

## Introduction

Neck pain and low back pain are currently the leading causes of years lived with a disability internationally [1]. The course of neck pain has been characterized as recurrent episodes occurring over a lifetime with variable degrees of recovery between episodes [2]. The past 20 years in particular has seen a surge in research into the way in which neck pain impacts on the cervical motor system, posture and movement. Motor output of cervical muscles is impaired. Muscles have decreased strength, endurance, force steadiness [3–6] and cervical muscle behaviour is altered eg., decreased activity of deep postural muscles, reduced directional specificity, delayed onset of muscle responses, muscle fatigability and increased neck muscle co-contraction [7–11]. Changes have also been documented in muscle morphology such as atrophy and fatty infiltrate [12, 13]. All functions of neck muscles can potentially be affected by neck pain. In recent years, changes in behaviour (activity) between deep and superficial neck muscles has been researched in both prescribed and functional tasks [8, 14, 15]. Most work to date has focussed on the neck flexors, the deep longus capitis and colli muscles and the superficial muscles, sternocleidomastoid (SCM) and anterior scalenes (AS) as they have some functional specificity in supporting the weight of the head, the cervical segments and cervical curve [16, 17]. The changed muscle behaviour in patients with neck pain presents as impaired (reduced) deep flexor muscle activity associated with increased activity in SCM and AS [8, 15, 18].

The place of exercise in rehabilitation programs for patients with neck pain disorders is well accepted. However even though research has shown a variety of changes in neuromuscular function, the type of the exercise intervention prescribed for persons with neck pain is often unidimensional and may focus either on strengthening, flexibility or motor control training [19]. The effectiveness of exercise programs is judged in most randomized controlled trials and attendant systematic reviews on changes in neck pain [19] and not on changes in physical functions. Although various changes in cervical motor output and muscle behaviour have been documented in association with neck pain, there has been no systematic review, which has focused on the effectiveness of an exercise intervention in terms of changing the various impaired physiological functions.

We focus on a commonly prescribed intervention, deep cervical flexor (DCF) training, to investigate differential physiological effects. DCF training is low load, without resistance and is performed in supine lying or other positions eg. sitting. The exercise intervention is based on research demonstrating that when persons with neck pain perform craniocervical flexion, they have reduced activation (electromyography (EMG)) of the deep flexors and greater (compensatory) activation of the SCM and AS when compared to persons without a neck pain disorder [8]. The craniocervical flexion test (CCFT) to detect this altered muscle behaviour is performed in a supine position. Guidance is provided by feedback from an inflatable pressure sensor inserted behind the neck - baseline pressure is 20 mmHg [20]. The person attempts to target five progressive positions of increasing range i.e. flexing to reach pressure increments of 22 mmHg, then to 24 mmHg and so forth to 30 mmHg. Laboratory measurements have used conventional surface EMG to record activity in the SCM and AS and a purpose constructed surface electrode inbuilt into a nasopharyngeal catheter to record activity of the deep cervical flexors [8, 21]. The latter is invasive and most clinical studies have measured the change in activity in SCM only as the outcome of the CCFT. An inverse relationship has been demonstrated between activity in the deep and superficial flexors during this test, ie. the lesser or poorer the activation of the deep cervical flexors, the higher the activation of SCM and AS [18]. The outcome of the CCFT can also be judged by which of the five pressure levels the participant can achieve using the movement of craniocervical flexion without palpably excessive use of the SCM or AS. This method is commonly used in the clinical setting [22].

This review is important to undertake as the true burden of neck pain is in its recurrent or persistent state. Comprehensive rehabilitation of all motor impairments could make a significant contribution to reducing the years lived with a disability and the associated personal, social and economic burden of neck pain. Thus it is vital to know what physiological functions DCF training can and cannot address. The objective of this study was to systematically review the literature using the exercise intervention of DCF training in patients with chronic neck pain to determine any evidence of its effectiveness in addressing impaired physiological functions, cervical neuromuscular function, muscle size, kinematics and kinetics.

## Method

### Design and search strategy

A systematic search of the literature was carried out to identify randomized controlled trials (RCT) written in English from four different databases; Web of Science, Scopus, CINAHL, and PubMed. The searches were performed from the inception until January 21st 2018 using the word “training” or “exercise” in combination with appropriate keywords to increase the breadth of the review. Keywords were combined without quotations marks or MESH terms. The following combinations of keywords were used in the search: Deep cervical flexor training or exercise; Craniocervical flexion training or exercise; Endurance training or exercise AND neck pain; Motor control training or exercise AND neck pain; Stabilization training or exercise AND neck pain; Neuromuscular training or exercise AND neck pain.

The specific search method for each database was:

- Web Of Science (WOS): Advanced search. Filter to “WOS ™ core collection”. TS = “keywords”. Refine by “articles” and “english” as language.
- Scopus: Basic search. Choose Field: “Article title, Abstract, Keywords”. Refine by “articles” and “english”.
- CINAHL: Advanced search. Refine by “peer reviewed”, “english language”, “research article”, “publication type: randomized controlled trial”, “language: english”
- PubMed: Basic search. Refine by “Randomized controlled trial” and “english” for language.

### Study selection

The databases were searched and duplicates removed. All titles and abstracts were screened independently by two authors (JB and ES) and consensus sought on which studies to obtain full texts for review. The full texts of these studies were assessed independently by JB and ES against inclusion and exclusion criteria. Exclusion criteria were applied in two steps as described below. If there were any disagreements they were resolved through discussion. If agreement was not reached, a third and fourth researcher (UR and GJ) was consulted to reach consensus.

#### Inclusion criteria

- Patients with neck pain disorders
- RCTs where DCF training was compared to another or no intervention
- DCF training was the sole intervention
- Outcomes were physiological measures of neck function

#### Exclusion criteria

##### Step 1

- RCTs using healthy, asymptomatic participants
- Case reports, reviews
- The training program used a combination of inventions precluding the evaluation of DCF training alone.
- Manuscript was in a language other than English

##### Step 2

- Poor quality of methods or reporting of outcomes of physiological measures precluded extraction of meaningful data.

### Risk of bias assessment

All five authors independently assessed risk of bias for the 12 studies included in the review using the Cochrane Collaboration Risk of Bias tool [23]. This instrument evaluates seven domains related to the validity of the study; random sequence generation, allocation concealment (selection bias), blinding of participants and personnel (performance bias), blinding of outcome assessment (detection bias), incomplete outcome data (attrition bias), selective reporting (reporting bias) and other bias (i.e., any bias not addressed by the above domains but of relevance for this current review). Evaluations in each domain are categorized as low risk of bias, high risk of bias or unclear. The category of unclear is used if the risk of bias can not be estimated due to lack of information [23]. Disagreement between the authors was discussed to reach consensus.

### Data extraction

The following data were extracted from the studies included in the review: participants characteristics (such as the number and specifications of participants (e.g. mechanical neck pain, whiplash), gender, age, duration, level of pain and self-rated functioning; type and duration of interventions in experimental and comparison groups; physiological outcome measures (including neuromuscular function, muscle size, kinetics, kinematics); pre to post intervention outcomes - within and between group effects on physiological measures. Data were extracted by two authors (JB and ES) who worked in consultation with authors (UR, GJ). All data extracted were finalized after discussion between all authors.

### Data analysis

All data were analysed qualitively and presented narratively in the result section. Meta- analyses were perfomed using the RevMan software (Cochrane group) for physiological outcome measures that were assessed in three or more studies. The data were retrieved either from the manuscripts or by contacting the authors. The results from the meta-analyses are presented in forest plots including statistical analysis of group differences and for heterogeneity, including p, chi^2^ and I^2^ values.

## Results

### Search and selection of studies

**Fig. 1:**
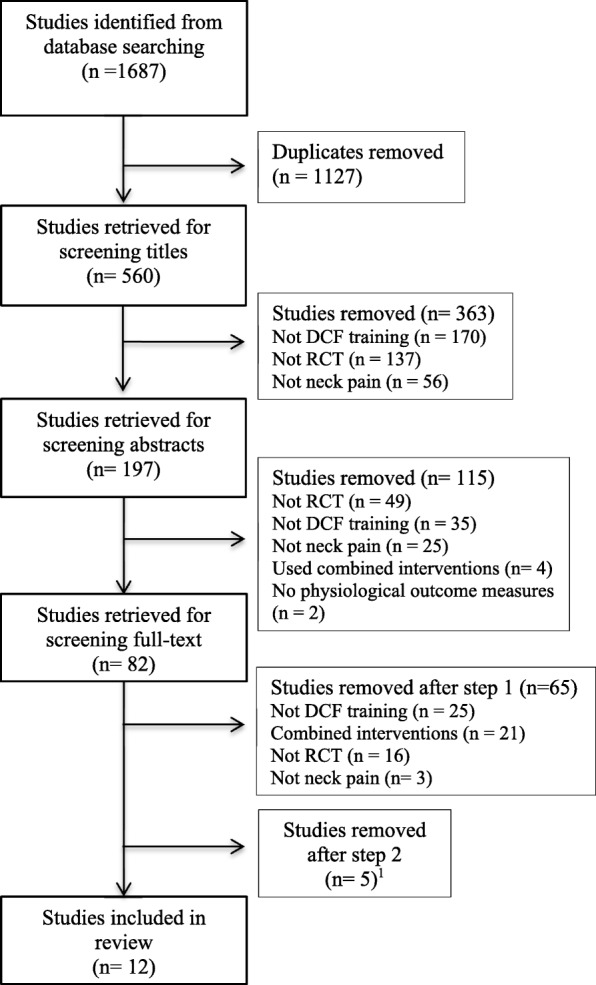
Flowchart for selection of the studies.^1^Reasons for exclusion: important information on how assessments of outcome variables and/or interventions were performed was missing; questionable validity of outcome measure

Figure 1 presents the study flow. The search of the databases yielded a total of 1687 studies of which 560 remained after duplicates were removed. After the screening of titles, a further 363 studies were removed and 197 abstracts were screened. The full text of 82 studies were retrieved and the first step of the screening process excluded 65 of these studies. The second step excluded a further five studies due to ambiguities in their method sections that made interpretation of methods used for assessment and/or intervention hazardous to interpret [24–28]. Four of these had unclear descriptions of the outcome measures [25, 26, 28] or used a questionable method for scoring of outcome measures [24]. One study [27] lacked description of the interventions. Authors of all five studies were contacted by e-mail with the aim to include the articles after eradicating the uncertainties. Only one author responded [24], but the issues were not resolved and the study remained excluded. A total of 12 studies were included in this review.

### Description of studies

The 12 studies involved a total of 502 participants with a persistent neck pain disorder (Table 1). Three studies used the same participant samples to measure different physiological effects [29–31]. Neck pain disorders were either of non-traumatic onset, specified as work-related [32] or non-specific [33, 34], a mix of traumatic (whiplash related) and non-traumatic onset [21, 29–31, 35–37] or not specified [38, 39]. The intervention periods varied from 2 weeks [38] to 12 weeks [32]. All interventions were evaluated directly after completion of the training program. One study included a follow-up at 26 weeks [37]. All studies included adults except one, for which high school students aged 17 years were recruited [33]. Eight studies included women only [21, 29–33, 35, 36]. The other four included both men and women [34, 37–39].

**Table 1:** Summary of reviewed studies

Study	Participants characteristics (gender, age, pain, function, duration of pain)	Intervention	Primary outcome	Results	Conclusions
		Intervention period Number of participants (n)			
Beer et al. 2012 [38]	Female (*n* = 10) Male (*n* = 10)) Age (y); Gr.1: 26.8 (±9.6) Gr.2: 31.7 (±13.3)	1. DCF training in sitting (postural correction exercise) (*n* = 10) 2. Control Group (no exercise) (*n* = 10)	Change in muscle activity in the CCFT (EMG) - SCM	CCFT (EMG) SCM - Gr 1 vs Gr 2: ♦ - Gr 1: + Gr 2: 0	DCF training in a postural correction exercise decreased SCM activity across all CCFT stages, but differences were significant only at the first and third stages of the test (22 and 26 mmHg levels). No change observed in the control group who did not exercise.
	VAS 0–10; Gr.1: 3.0 (±1.7) Gr.2: 2.6 (±1.1)	Intervention period: 2 weeks			
	NDI 0–100; Gr.1:18.1 (±9.0) Gr.2: 20.6 (±10.1)				
	Duration of pain (y); Gr.1: 4.7 (±3.3) Gr.2: 7.9 (±9.0)				
Borisut et al. 2013 [32]	Females (*n* = 100) Age (y); Gr.1: 32.7 (±3.1) Gr.2: 30.4 (±3.4) Gr.3: 30.2 (±3.0) Gr.4: 29.3 (±3.1)	1. DCF training (*n* = 25) 2. Neck flexor, extensor strength and endurance training (*n* = 25) 3. Combined DCF, strength, endurance training (*n* = 25) 4. Control group (*n* = 25)	Cervical muscle activation (RMS EMG amplitude) during a sitting typing task - Upper trapezius (UTr) - Cervical erector spinae (ES) - Sternocleidomastoid (SCM) - Anterior scalenes (AS)	Muscle activation (EMG):	All exercise intervention groups (Gr’s 1, 2, 3) similarly and significantly reduced muscle activity in all muscles evaluated in the typing task with the exception of the (L) ES. No exercise group had a superior effect.
				(R) (L) UTr -Gr 1 vs 4: ♦ Gr 1 vs 2,3: 0	
				(R) (L) SCM -Gr 1 vs 4: ♦ Gr 1 vs 2,3: 0	
				(R) (L) AS - Gr 1 vs 4: ♦ Gr 1 vs 2,3: 0	
				(R) ES - Gr 1 vs 4: ♦ Gr 1 vs 2,3: 0	
				(L) ES - Gr 1 vs 2,3,4: 0	
	VAS 1–100; Gr.1: 55.0 (±11.0) Gr.2: 56.0 (±22.7) Gr.3: 61.5 (±16.7) Gr.4: 59.0 (±10.5)	Intervention period: 12 weeks		- Gr 1: + All muscles Gr 2: + All muscles Gr 3: + All muscles Gr 4: 0 All muscles except UTr	No changes in EMG activation occurred in the control group except for UTr where, in contrast to the interventions, muscle activity increased.
	NDI ; Gr.1: 28.2 (±5.6) Gr.2: 30.0 (±4.5) Gr.3: 29.2 (±5.3) Gr.4: 31.6 (±5.1)				
	Duration of pain (months); ≥6.				
Falla et al. 2006 [29]	Females (*n* = 58). Age (y); Gr.1: 37.7 (±9.9) Gr.2: 38.1 (±10.7)	1.DCF training (*n* = 29) 2. Cervical flexor endurance-strength training (*n* = 29)	Comparison of effect of each exercise mode on: Fatigability of cervical flexors, SCM, AS (EMG) - Mean spectral frequency MSF - Average rectified value ARV - Conduction velocity CV	Fatigability (EMG) MSF: - Gr 1 vs 2: ★ - Gr 1: 0 Gr 2: +	DCF training did not improve any measures of superficial cervical flexor fatigability.
					Endurance-strength training significantly improved fatigability measures of MSF and ARV, but had no effect on CV.
				ARV: - Gr 1 vs 2: ★ - Gr 1: 0 Gr 2: +	
	VAS 0–10; Gr.1: 3.6 (±2.0) Gr.2: 4.7 (±2.0)	Intervention period: 6 weeks			
				CV: - Gr 1: 0 Gr 2: 0	
			Strength (MVC)		Endurance-strength training was superior in improving strength of the cervical flexors compared to DCF training.
	NDI 0–50; Gr.1: 9.8 (±3.3) Gr.2: 10.4 (±3.4)				
				Strength (MVC) - Gr 1 vs Gr 2 ★ - Gr 1: 0 Gr 2: +	
	Duration of pain (y); Gr.1: 7.5 (± 5.9) Gr.2: 8.3 (± 7.0)				
Falla et al. 2007 [30]	Females (*n* = 58) Age (y); Gr.1: 37.7 (±9.9) Gr.2: 38.1 (±10.7)	1. DCF training (*n* = 29) 2. Cervical flexor endurance-strength training (*n* = 29)	Change in postural angle during a 10 min computer task - Cervical angle (line between C7 and tragus to horizontal - forward head position) - Thoracic angle (line between C7 and T7 to horizontal - upper thoracic flexion posture)	Change in postural angle: Cervical angle - Gr 1 vs Gr 2: ♦ - Gr 1: + Gr 2: 0	DCF training resulted in a lesser change towards a forward head posture during the 10 min computer task. No change was evident in the endurance-strength training group.
	VAS 0–10; Gr.1: 3.6 (± 2.0) Gr.2: 4.7 (± 2.0)	Intervention period: 6 weeks			
				Thoracic angle - Gr 1 vs Gr 2: 0 - Gr 1: + Gr 2: +	Both DCF and strength-endurance training reduced the change in thoracic flexion angle during the 10 min computer task with no difference between groups.
	NDI 0–50; Gr.1: 9.8 (± 3.3) Gr.2: 10.4 (± 3.4)				
	Duration of pain (y); Gr.1: 7.5 (± 5.9) Gr.2: 8.3 (± 7.0)				
Falla et al. 2008 [31]	Females (*n* = 58) Age (y); Gr.1: 37.7 (±10.1) Gr.2: 38.1 (±10.7)	1. DCF training (*n* = 29) 2. Cervical flexor endurance-strength training (*n* = 29)	Cervical muscle activation (RMS EMG amplitude) during a pencil tapping task - SCM (L) and (R)	Muscle activation (EMG): (R) (L) SCM - Gr 1 vs Gr 2: 0 - Gr 1: 0 Gr 2: 0	Neither DCF training nor cervical flexor strength-endurance training translated to a change in SCM muscle activity in a functional pencil tapping task.
	VAS 0–10; Gr.1: 3.5 (±2.0) Gr.2: 4.7 (±2.0)	Intervention period: 6 weeks			
	NDI 0–50); Gr.1: 9.8 (±3.3) Gr.2: 10.1 (±3.0)				
	Duration of pain (y); Gr.1: 7.6 (± 6.0) Gr.2: 8.3 (± 7.0)				
Ghaderi et al. 2017 [39]	Females and males (*n* = 40). Age (y); Gr.1: 36.0 (±2.5) Gr.2: 36.3 (±3.1)	1. DCF training (*n* = 20) 2. Progressive resistive isometric exercises 30% MVC and neck postural control exercises (*n* = 20)	Comparison of effect of each exercise mode on: Change in muscle activity in the CCFT (EMG) - SCM - AS - SC	CCFT (EMG) - (R) (L) SCM, AS & SC (Gr 1 vs 2): ♦ - Gr 1: + Gr 2: -	DCF training decreased SCM, AS and SC EMG during the CCFT compared to isometric exercise group.
				Relative latency neck muscle onset (EMG) - Not clear from paper if difference between groups or not. - Gr 1: 0 Gr 2: +	Decreased relative latency of the superficial neck muscle during rapid arm movements were reported for both the DCF training and isometric training groups but differences were reported as significant only for the isometric training group.
	VAS 0–100; Gr.1: 61.4 (±28.0) Gr.2: 59.7 (±22.7)				
			Relative latency of superficial neck muscle onset compared to DA during rapid unilateral arm movements (EMG)		
				Endurance time DCF - Gr 1 vs Gr 2: 0 - Gr 1: + Gr 2: 0	
					Endurance time increased significantly for the DCF training group but not the isometric training group. There was no significant difference between groups.
	NDI 0–100; Gr.1: 31.3 (±12.1) Gr.2: 34.3 (±14.3)	Intervention period: 10 weeks	Endurance time of DCF muscles in CCFT		
	Duration of pain (week); Chronic neck pain >12w				
Javanshir et al. 2015 [34]	Females (*n* = 40) Males (*n* = 20). Age (y); Gr.1: 36.8 (±3.5) Gr.2: 35.7 (±5.0)	1.DCF training (*n* = 30) 2.Cervical flexor strength training (*n* = 30)	Comparison of effect of each exercise mode on muscle dimensions (ultrasonography) -Longus colli -SCM	Muscle dimensions Longus colli: - Gr 1 vs 2: ♦ - Gr 1: + Gr 2: 0	DCF training significantly increased the size of longus colli but failed to change the dimensions of the SCM significantly.
				SCM: - Gr 1 vs 2: ★ - Gr 1: 0 Gr 2: +	In contrast, cervical flexor training significantly increased the size of SCM but did not have significant effects on longus colli dimensions.
		Intervention period: 10 weeks			
	VAS 0–10; Gr.1: 5.0 (±2.4) Gr.2: 5.1 (±2.2)				
	NDI 0–100; Gr.1: 33.3 (±11.4) Gr.2: 33.2 (±15.0)				
	Duration of pain (y); Gr.1: 3.3 (±3.2) Gr.2: 3.3 (±3.2)				
Jull et al. 2007 [35]	Females (*n* = 58) Age (y); Gr.1: 42.7 (±10.8) Gr.2: 39.0 (±11.6)	1.DCF training (*n* = 30) 2.Proprioception training (*n* = 28)	Comparison of effect of each exercise mode on cervical proprioception. Measure of joint position error (JPE) on return to the natural head posture from: - Rotation (left and right) - Extension	Proprioception (JPE) Right Rotation - Gr 1 vs Gr 2: ★ - Gr 1: + Gr 2: +	Both DCF training and proprioception training resulted in a significant decrease in JPE (improvement) compared to baseline in all the three movement directions.
		Intervention period: 6 weeks			
	NRS 0–10; Gr.1: 6.3 (±1.7) Gr.2: 6.7 (±1.5)				
				Left Rotation - Gr 1 vs Gr 2: 0 - Gr 1: + Gr 2: +	There were no significant differences in gains made by the exercise interventions. The exception was from right rotation where the reduction in JPE was significantly greater with proprioception training.
	NDI 0–50; Gr.1: 17.6 (±4.9) Gr.2: 21.6 (±9.1)				
				Extension - Gr 1 vs Gr 2: 0 - Gr 1: + Gr 2: +	
	Duration of pain (y); Gr.1: 8.7 (±7.1) Gr.2: 10.6 (±9.3)				
Jull et al. 2009 [21]	Females (*n* = 46) Age (y); Gr.1: 39.6 (±12.2) Gr.2: 37.1 (±10.3)	1.DCF training (*n* = 23) 2. Cervical flexor strength training (*n* = 23)	Comparison of effect of each exercise mode on:	CCFT (EMG) Longus capitis/colli - Gr 1 vs Gr 2: ♦ - Gr 1: + Gr 2: 0	DCF training resulted in a significant increase in EMG amplitude of longus capitis/colli and a significant decrease in SCM and AS activity in the CCFT. No significant changes were observed with cervical flexor strength training.
			CCFT (EMG) - Longus capitis/colli - SCM - AS		
				SCM: - Gr 1 vs Gr 2: ♦ - Gr 1: + Gr 2: 0	
	NRS 0–10; Gr.1: 4.5 (±1.6) Gr.2: 4.2 (±2.1)	Intervention period: 6 weeks			
					Neither intervention changed the total ROM used in the CCFT, but DCF training resulted in a significant relative increase in the ROM used in each stage of the test.
				AS: - Gr 1 vs Gr 2: ♦ - Gr 1: + Gr 2: 0	
			Range of movement in the CCFT CCF ROM (full) CCF ROM at each stage		
	NDI 0–50; Gr.1: 11.0 (±2.7) Gr.2: 9.6 (±3.1)				
	Duration of pain (y); Gr.1: 10.1 (±10.6) Gr.2: 9.2 (±6.6)		Relative latency of neck flexor muscle onset in flexion and extension arm movement tasks (EMG) - Longus capitis/colli - SCM - AS	CCF ROM (full): - Gr 1: 0 Gr 2: 0 CCF ROM (stages): - Gr 1 vs Gr 2: ♦ - Gr 1: + Gr 2: 0	There was no significant change in the relative latencies of EMG onsets in any cervical muscle during unilateral arm movements with either training regime.
					When data were analysed in 10 s epochs, the proportion of participants who showed earlier onsets in the longus capitis longus/colli was significantly greater in the DCF training group compared to the strength training group. A greater proportion of subjects showed earlier onsets
				Relative latency neck flexors (EMG) Longus capitis/colli, SCM, AS - Gr 1 vs Gr 2: 0 - Gr 1: 0 Gr 2: 0	
				Longus capitis/colli (proportions achieving earlier onsets) - Gr 1 vs Gr 2: ♦ - Gr 1: + Gr 2: 0	
Lee et al. 2013 [33]	Females (*n* = 30) Age (y): m = 17	1. DCF training (*n* = 15) 2. Control group – Basic stretching exercise (*n* = 15)	Comparison of effect of each exercise mode on:	CCFT - Gr 1 vs Gr 2: ♦ - Gr 1: + Gr 2: 0	DCF training resulted in significantly improved performance in the CCFT and in the neck-shoulder postural parameters measured.
	NDI 0–50; < 15		CCFT Clinical assessment of test level achieved		
	Duration of pain (months); ≥ 3.	Intervention period: 8 weeks		Neck-shoulder posture HTA; NFA, FSA - Gr 1 vs Gr 2: ♦ - Gr 1: + Gr 2: 0	Basic stretching exercises did not have a significant effect on either CCFT performance or posture.
			Neck-shoulder posture - HTA (head tilt angle) - NFA (neck flexion angle) - FSA (forward shoulder angle)		
O’Leary et al. 2007 [36]	Females (*n* = 50) Age (y): Gr.1: 36.9 (±9.5) Gr.2: 37.9 (± 11.3)	1. DCF training (*n* = 27) 2. Cervical flexion (head lift) exercise (*n* = 23)	Comparison of effect of each exercise mode on:	Craniocervical flexor strength (MVC) - Gr 1 vs 2: 0 - Gr 1: + Gr 2: +	There were no significant differences between groups in exercise outcomes. DCF and head lift training both increased craniocervical flexor strength (by 11 and 12.2% respectively).
			Strength (MVC) - craniocervical flexors Endurance (time to failure 50% MVC) - craniocervical flexors		
				Craniocervical flexor endurance (MVC_50_) - Gr 1 vs 2: 0 - Gr 1: + Gr 2: +	
	NDI 0–100 (range); 10–28.	Intervention period: 6 weeks			
	Duration of pain (months); > 3.		Contraction accuracy (force steadiness) - Percentage of recording remaining within ±3% of torque task	Contraction accuracy - Gr 1 vs 2: 0 - Gr 1: + Gr 2: +	DCF and head lift training both increased craniocervical flexor endurance at MVC_50_ (time to failure increased by 37 and 16% respectively).
					DCF and head lift training both improved contraction accuracy (by 7 and 9% respectively).
^a^ O’Leary et al. 2012 [37]	Females (*n* = 35) Males (*n* = 25) Age (y); Gr.1: 37.8 (±12.6) Gr.2: 38.2 (±12.8) Gr.3: 37.7 (±12.7) VAS 0–100; Gr.1: 33.2 (±13.0) Gr.2: 29.9 (±14.5) Gr.3: 30.6 (±12.1) NDI 0–50: Gr.1: 9.8 (±2.1) Gr.2: 11.0 (±2.2) Gr.3: 10.5 (±2.5) Duration of pain (y); Gr.1: 7.1 (±4.6) Gr.2: 7.2 (±8.2) Gr.3: 6.2 (±5)	1. DCF training (*n* = 20) 2. Craniocervical flexor endurance training 20% MVC for 10 weeks progressed by time. For 5 weeks, brief holds at 50% MVC were added. (*n* = 20) 3. Active cervical mobility training (*n* = 20) Intervention period: 10 weeks; 26 week follow up.	Comparison of effect of each exercise mode on: CCFT: SCM and AS muscle activity (RMS EMG amplitude) in each of the five stages of the test Craniocervical flexor muscle endurance – Time to failure Craniocervical flexor muscle strength – Torque Cervical range of motion (CROM) - Flexion - Extension - Rotation	CCFT Muscle activation (EMG) Sternocleidomastoid (SCM) 10 weeks - Gr 1 vs 2: ♦ Gr 1 vs 3: ♦ Gr 2 vs 3: 0 26 weeks- Gr 1 vs 2: 0 Gr 1 vs 3: 0 Gr 2 vs 3: 0 Anterior scalene (AS): 10 weeks - Gr 1 vs 2: ♦ Gr 1 vs 3: 0 Gr 2 vs 3: 0 26 weeks- Gr 1 vs 2: 0 Gr 1 vs 3: 0 Gr 2 vs 3: 0 CCF Endurance 10 weeks - Gr 1 vs 2: ★ Gr 1 vs 3: 0 Gr 2 vs 3: ★ 26 weeks- Gr 1 vs 2: 0 Gr 1 vs 3: 0 Gr 2 vs 3: ★ CCF Strength10 weeks - Gr 1 vs 2: 0 Gr 1 vs 3: 0 Gr 2 vs 3: 0 26 weeks- Gr 1 vs 2: 0 Gr 1 vs 3: 0 Gr 2 vs 3: 0 Cervical range of motion10 weeks - Gr 1 vs 2: 0 Gr 1 vs 3: 0 Gr 2 vs 3: 0 26 weeks- Gr 1 vs 2: 0 Gr 1 vs 3: 0 Gr 2 vs 3: 0	Muscle activation (EMG) DCF training resulted in significant changes (decreases) in SCM activity in the CCFT compared to endurance training and mobility training at 10 weeks at the 24-30 mmHg stages of the CCFT. At 26 weeks, the changes with DCF training regressed and were not significantly different from the endurance and mobility training groups. DCF training resulted in significant changes (decreases) in AS activity in the CCFT compared to endurance training and mobility training at 10 weeks only at the 28 mmHg stage of the CCFT. At 26 weeks, there were no differences in changes with DCF training compared to the endurance and mobility training groups. Craniocervical flexor endurance DCF training resulted in no significant improvements in time to failure. Significant improvements were recorded for the endurance training group compared to the mobility group at both 10 and 26 weeks and compared to the DCF group at 10 weeks. Craniocervical flexor strength The endurance training group showed significant improvements in strength pre to post intervention at 10 and 26 week follow up. There were no significant differences in improvements in strength between groups. Cervical range of motion Very small gains in ROM were recorded, but there were no significant between group differences.

*Abbreviations*: ♦ = DCF training significantly different/superior to a control or comparator group. + = DCF training or comparator significantly improved pre to post. 0 = DCF training or comparator, no significant difference between or within groups. - = DCF training or comparator significantly inferior pre to post. ★ = Control or comparator group significantly different/superior to DCF training or another comparator group.

^a^ Author contacted and data was reanalysed using change scores

DCF training was compared to either one or two other training regimes and/or a control group with no intervention. The comparator training regimes included cervical endurance training at progressive intensities of effort [37, 39] endurance-strength training [21, 29–32, 36] proprioception training [35], mobility training [37] and muscle stretching [33]. Two studies included a control group with no intervention [32, 38].

DCF training was performed in supine lying in all studies except two. In one, training was performed in sitting [38] and in the other, DCF training was undertaken in supine and in different (unspecified) positions [39]. In relation to physiological outcome measures, cervical neuromuscular function was measured via deep and superficial muscle behaviour (EMG amplitudes) in the CCFT [21, 33, 37–39], muscle activity (EMG amplitudes) in functional tasks (manual work in a sitting posture) [31, 32], muscle onsets during rapid arm movements [21, 39], and muscle fatigability during submaximal endurance tasks [29]. Other outcome measures included cervical muscle size [34], kinematic measures of cervical joint position sense (JPS) [35], head and spinal posture [30, 33] and cervical range of motion [21, 37], as well as kinetic measures of maximum cervical muscle force [29, 36, 37], endurance [36, 37, 39] and force steadiness [36].

### Risk of bias

**Fig. 2:**
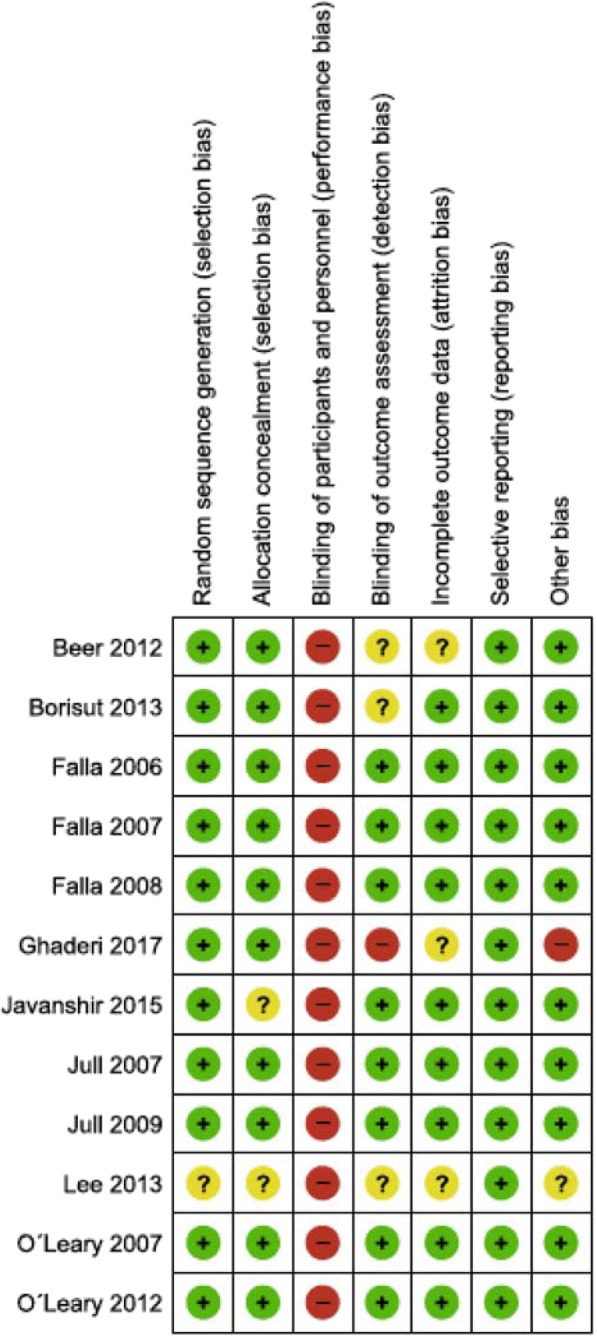
Risk of bias of included studies

The summary of the risk of bias assessment is presented in Fig. 2. Selection bias: The methods reported for random sequence generation were assessed to be at low risk in 11 studies. One study [33] was rated unclear due to lack of information. Allocation concealment was assessed as unclear in two studies. In one, allocation concealment was not specified [33], and in the other, the size of block randomization not described [34]. Performance and detection bias: All 12 studies were judged to be at high risk for performance bias. However, considering the inherent nature of the exercise interventions provided, blinding of practitioners and participants was not possible. Whether or not blinding of outcome assessment occurred was unclear in three studies [32, 33, 38]. One study [39] explicitly stated that the study was not blinded and therefore rated high risk for detection bias. Attrition bias: three studies [33, 38, 39] did not provide enough information to make judgement about the risk of bias of incomplete outcome data. Other bias: two studies were judged to have other sources of bias, due to unclear descriptions of the position in which the DCF training was conducted [39] and due to insufficient description of measurements [33, 39]. Overall, seven studies [58%] were assessed to be at low risk of bias across six of the seven domains [21, 29–31, 35–37]. Two studies are prone to a higher risk of bias compared to the others one study was rated unclear in five of the seven domains [33] and the other was rated as high risk of bias in three of the seven domains [39].

### Effects of the intervention

Table 1 summarises the effects of the interventions on the various outcome measures of physiological functioning.

### Neuromuscular function

#### Craniocervical flexion test (CCFT)

##### Performance

One study investigated performance in the CCFT, i.e. participants’ ability to reach each of the five test stages without compensatory movement [33]. The results showed that the DCF training group achieved a significant increase (improvement) in the test stages achieved pre to post intervention. No change in performance was recorded for the control group who performed stretching exercises.

##### EMG amplitude

Four studies investigated EMG amplitude of neck muscles during the CCFT [21, 37–39]. Muscles investigated were superficial cervical flexor muscles SCM [21, 37–39] and AS [21, 37–39], the superficial extensor muscle splenius capitis (SC) [39], and the deep craniocervical flexor muscles (longus capitis, longus colli) [21]. All studies consistently showed a significant reduction (desired) in SCM, AS and SC EMG amplitude during the performance of the CCFT following DCF training pre to post intervention (within group) as well as between group differences with comparator training. A significant reduction was also seen within the DCF training group, but not between groups, at 26 weeks follow up in one study [37]. No significant reduction was reported in superficial cervical muscle activity in the comparator groups, which tested strength training, endurance training, active mobility training or no training. One study reported a significant pre to post intervention increase (desired) in the craniocervical flexor muscle (longus capitis/colli) EMG amplitude following DCF training compared to strength training which achieved no significant change [21].

**Fig. 3:**
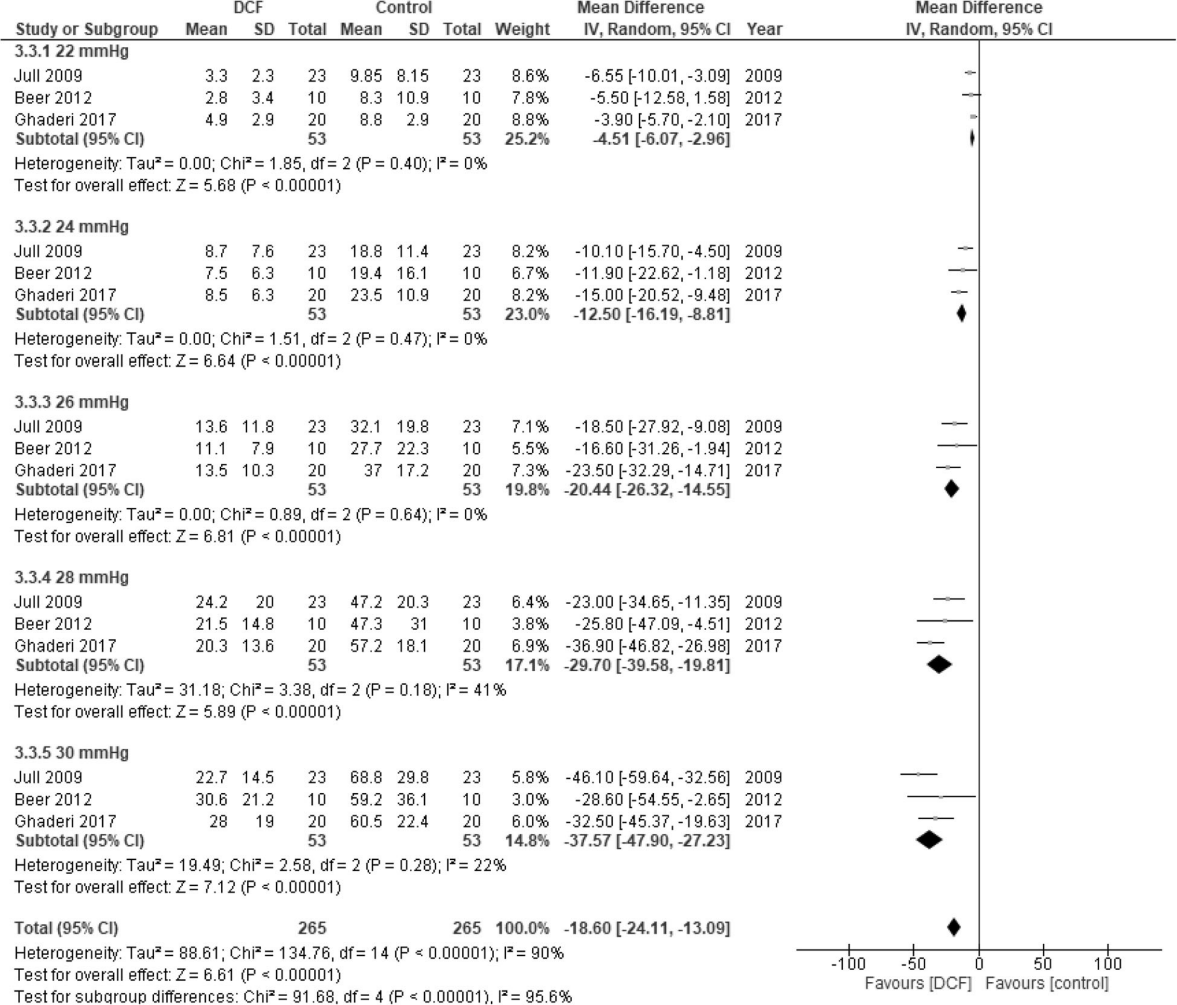
Forest plot of meta-analysis comparing DCF training with strength-endurance training (Jull et al. 2009 [21] and Ghaderi et al. 2017 [39]) and no intervention (Beer et al. 2012 [38]) on the effects of RMS EMG of sternocleidomastoid (SCM) during the craniocervical flexion test (CCFT). The mean and standard deviation (SD) are the values from the post intervention measures. Raw data was supplied from Beer et al. 2012 [38] while all other data was extracted from the original studies. Average of the EMG data from the left and right SCM was used for data analysis

A meta-analysis was performed to evaluate the effects on EMG amplitude for each of the five stages of the CCF test, 22 mmHg to 30 mmHg, for SCM. Unfortunately, we could not extract the required data from one of the studies [37] and the author responsible for the data analyses was not reachable. We therefore could not access the raw data which precluded this study from the meta analysis. The analysis therefore includes three studies comparing DCF training with strength-endurance [21, 39] or no intervention [38]. The results show a significant reduction in SCM muscle activity in favour for DCF training compared to control groups. This is consistent for each of the five levels and for the total of all levels (Fig. 3).

#### Functional task

##### EMG amplitude

Two studies investigated the EMG amplitude of cervical muscles during a sitting, light manual task [31, 32]. Muscles investigated were SCM [31, 32], AS, cervical erector spinae and upper trapezius [32]. Borisut et al. [32] reported that all training interventions, i.e. DCF training, strength-endurance training and combined DCF and strength-endurance training, significantly reduced pre to post intervention EMG amplitude during a typing task, a desired outcome. There were no significant differences between training groups, but all training groups were significantly different to the control group (no intervention) for all muscles [32]. Falla et al. [31] in contrast, showed no significant change in SCM EMG amplitude pre to post intervention for either the DCF training group or strength-endurance group during a repetitive pen and paper task.

##### EMG onset

Two studies evaluated the relative latency (EMG onset) of the neck muscles relative to the deltoid muscle during rapid arm movements [21, 39]. One study evaluated the flexor muscles (the deep craniocervical flexors and SCM and AS) [21]. An earlier, but non-significant, onset of the deep craniocervical flexors was seen for the DCF training group compared to the strength training group [21] post intervention. However, significantly more participants in the DCF training group showed a desired earlier onset of the DCF relative to the deltoid muscle after the intervention. Ghaderi et al. [39] evaluated the superficial neck flexor muscles SCM and AS and the extensor muscle SC. Latency decreased in all muscles following both the DCF training and isometric resistive exercise groups but the differences were significant in the isometric resistive exercise group only.

#### Muscle fatigability

##### EMG fatigue

One study evaluated fatigability (EMG) of SCM and AS muscle during submaximal cervical flexion contractions (MVC_10_, MVC_25_ and MVC_50_) [29]. DCF training had no significant effect on fatigability but significant improvements were reported for endurance-strength training compared to DCF training for the fatigability measures - mean spectral frequency and average rectified value for both SCM and AS muscles.

#### Muscle size

##### Cross sectional area, width and thickness

One study [34] evaluated the dimensions of the longus colli and SCM muscles with ultrasound imaging before and after DCF training and cervical flexor strength training. DCF training resulted in a significant increase in the dimensions of longus colli (cross sectional area, width and thickness) compared to strength training. There was no change in SCM thickness in the DCF training group. Instead strength training significantly increased SCM thickness compared to DCF training.

#### Kinematics

##### Joint position sense (JPS)

One study [35] compared DCF training and cervical proprioception training to evaluate effects on a proprioception measure of JPS following active movement from right and left neck rotation and extension. Both the DCF training and proprioception training groups showed a significant post intervention improvement in JPS compared to baseline in all movement directions. However, the proprioception training group showed a significantly larger improvement on return from right rotation compared to the DCF training group.

##### Posture

Two studies investigated the effects of training on sitting posture [30, 33]. Both studies found that DCF training was effective in improving posture. Lee et al. [33] measured head, neck and shoulder posture by comparing three different angles on X-ray; head tilt angle, neck flexion angle and forward shoulder angle. They determined a significant improvement in posture in all three angles following DCF training compared to basic stretching exercises for the neck and shoulder which showed no change. Falla et al. [30] used a digital photographic technique to measure any progressive changes in cervical (forward head posture) and upper thoracic posture during a 10 min computer task. The DCF training resulted in a significant reduction in the change of cervical angle (reduced forward head posture) compared with the endurance-strength training. Both groups improved their ability to maintain an upright posture of the thoracic spine with no significant difference between the groups.

##### Range of motion

Cervical range of motion (ROM) was evaluated in two studies [21, 37]. One study compared the effects of DCF training, active mobility training and endurance training on ROM [37]. A 3D motion-tracking device was used to measure cervical flexion, extension and rotation left and right. There was a small effect of time on ranges of flexion and left rotation but training mode did not significantly affect outcome. Jull et al. [21] evaluated craniocervical ROM using a digital imaging method to record total craniocervical ROM as well as ROM in each stage of CCFT. No significant difference was observed in total craniocervical ROM used by either group post intervention. However, a significant improvement in relative ROM was seen following DCF training compared to strength training. DCF training improved range at all five stages of the CCFT, while the strength training group improved only at two test stages.

#### Kinetics

##### Strength

Three studies evaluated strength of cervical flexor [29] or craniocervical flexor muscles [36, 37] by measuring maximum voluntary isometric contraction (MVIC) with dynamometers. Falla et al. [29] found that endurance-strength training resulted in significantly greater improvements in cervical flexor strength. No changes in strength were observed in the DCF training group. O’Leary et al. [36] determined no difference in craniocervical muscle strength between DCF training and cervical flexor endurance training (head lift exercise) with a 12 and 11% gain respectively. In a second study, craniocervical flexor muscle endurance training predominantly at 20% MVC achieved significant improvements in craniocervical muscle strength but, although greater than those achieved by DCF training and mobility exercises, the difference was not significant [37].

**Fig. 4:**
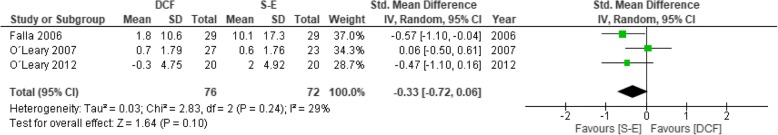
Forest plot of meta-analysis comparing DCF training with strength-endurance training on the effects of cervical muscle strength. The mean and standard deviation (SD) are changes in values between baseline and post intervention measures. Data from Falla et al. 2006 [29] was used to impute the SD values for O’Leary et al. 2007 [36] and for O’Leary et al. 2012 [37] as described by Cochrane handbook chapter 16.1.3.2

A meta-analysis including the three studies comparing DCF training with various strength-endurance training regimes was performed to evaluate the effects on strength [29, 36, 37]. Results showed a tendency in favour of strength-endurance training but did not reach significant differences (*p* = 0.10) (Fig. 4).

##### Endurance

Three studies evaluated endurance of the DCF muscles [36, 37, 39]. One study reported significantly longer holding time during CCFT in the DCF training group. There was a tendency for better improvement with DCF training compared to isometric resistive training group but the difference was not significant [39]. Two studies evaluated craniocervical muscle endurance measuring time to task failure for sustained contractions of the craniocervical flexors at 50% maximal voluntary contraction (MVC_50_) using a dynamometer [36, 37]. O’Leary et al. [36] reported significant improvement in craniocervical muscle endurance pre to post intervention with both DCF training and cervical flexor endurance training (head lift exercise) with no significant between group differences. In a second study, craniocervical flexor muscle endurance training predominantly at 20% MVC (MVC_20_) achieved significant improvements in craniocervical muscle endurance. The improvements were significantly greater than those achieved with DCF training and mobility exercises at the 10 week follow-up [37]. The improvement remained significant for endurance training compared to mobility training at the 26 week follow-up, but just failed to reach significance compared to DCF training.

**Fig. 5:**
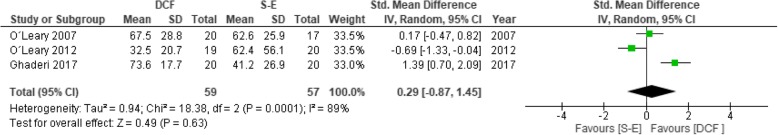
Forest plot of meta-analysis comparing DCF training with strength-endurance training on the effects of cervical muscle endurance. The mean and standard deviation (SD) are the values from the post intervention measures. All values were extracted from the original studies

A meta-analysis including the three studies comparing DCF training with various strength-endurance training regimes [36, 37, 39] was performed to evaluate any overall differences between training regimes. There was a large variation between studies regarding effects on the endurance measures, as presented above, and the results showed no significant differences between exercise regimes (Fig. 5).

##### Contraction accuracy (force steadiness)

One study [36] determined that both DCF training and cervical flexor strength training significantly improved contraction accuracy (ability of maintain a contraction at 50% MVC within ±3% of the expected torque task). There were no significant differences between training groups.

### Adverse effects

Six of the 12 studies reported the occurrence or not of any adverse effects [29–31, 33, 36, 37]. Five of six studies reported no adverse effects [29–31, 33, 36]. In the study reporting an adverse affect, a participant withdrew due to symptom aggravation during a mobility training program [37]. The remaining six studies [21, 32, 34, 35, 38, 39] did not report data on adverse effects.

## Discussion

Neck pain is a recurrent disorder and comes with enormous personal, social and financial costs [1]. It is accompanied by an array of changes in the neuromuscular and sensorimotor systems [7–11] which result in a variety of impaired physiological functions. Exercise is a cornerstone of rehabilitation and a desired outcome is to reverse the impaired physiological functions towards prevention of recurrent episodes. Exercise programs often focus predominantly on one mode of training. The question was whether one mode of training could successfully address the different impaired physiological functions. This review used DCF training, a low load motor control training program, to systematically examine the evidence for which physiological functions this single mode of training could and could not address in persons with neck pain disorders.

The 12 studies included in this review trialed a number of exercise modes as the comparator to DCF training and measured a variety of physiological functions. In relation to neuromuscular coordination, evidence from four studies [21, 37–39] found that DCF training was more effective in addressing altered muscle behaviour by reducing activity in the superficial muscles SCM and AS and increasing activity in the deep craniocervical flexors [21] as well as improving performance in the CCFT [33]. Little if any change in muscle behaviour was achieved with the comparators of strength, endurance, flexibility or no training. This specificity of DCF training was also reflected in the study measuring muscle size [34] where this training selectively increased the size dimension of longus capitis/colli, an outcome not achieved with strength training.

The picture is less clear when considering translation of training effects to function. Two studies investigated the effects of DCF and strength training on cervical muscle activity during light functional tasks in sitting. One found that both training methods, similarly and desirably, reduced muscle activity [31, 32]. The other found that neither training method had an effect [31, 32]. Two studies measured cervical muscle onsets relative to deltoid muscle in arm movement tasks. In agreement, both found that both DCF and strength training reduced latency, but one study found that DCF training was more efficient [21] and the other that strength training was more efficient [39]. In contrast, there was agreement in findings of two studies which found that DCF training improved control of head and neck posture where as strength training and stretching exercises did not [30, 33].

For the functions of cervical muscle strength and fatigability the included studies not surprisingly indicates that DCF training is largely ineffective and training with load or resistance is required to increase muscle strength. Strength training appears to have greater effect on SCM than the craniocervical flexors [34] which could account for this result. It should be noted that the meta-analysis on endurance and also strength measures did not show significant differences between DCF training and various protocols for strength-endurance training. This may be due to the large variation between both assesment methods and comparative interventions. Our results show an indication, althought not significant (*p* = 0.1), in favour strength-endurance training to increase strength compared to DCF training. Future studies are needed to confirm or reject this indication. Nevertheless improvement in contraction accuracy occurred similarly with DCF and strength training. Regarding proprioception, DCF training was effective in improving JPS from extension and rotation but the specific proprioception program proved more effective notably from rotation [35]. When it comes to improve mobility there was no differential effect of training mode whether DCF, active mobility or endurance training on cervical range of motion [37].

### Limitations of the study

The results of this systematic review should be interpreted in light of some limitations. First, a number of physiological functions have been investigated in response to DCF training. Several studies have examined the effect on neuromuscular coordination between deep and superficial cervical flexors and cervical muscle strength-endurance, but other functions have been considered in one or two studies only, which severely limits any conclusion of effect for these functions. Second, measurements and their methods varied across studies and this heterogeneity precluded definite conclusion of the results from the meta-analyses on strength and endurance. Third, most studies were categorized as low risk of bias which can be seen as a strength. However at least two studies had higher risk of bias. All studies were rated as high risk of bias in their blinding of practitioners and participants. This is a common problem in exercise trials as it is impossible to blind the participants and the practitioners delivering the exercise from its type. Fourth, eight of the 12 studies included in the review [21, 29–31, 35–38] were performed in one research laboratory. One author of this review (GJ) was a member of that laboratory. Nevertheless, the outcomes of studies in other laboratories were not different except in the one instance of conflicting results for the translation of training to a functional task [29, 30]. The tasks were different between studies which may have influenced outcomes. Fifth, only one study investigated long term effects of DCF training [37] severely limiting any comment on maintinance of effect of exercise. Finally, only studies published in English were retrieved which possibly excluded relevant articles in other languages.

Further research is required to understand the specificity of effect of DCF training and indeed the effects of other modes of exercise. There is relatively strong evidence that DCF training addresses neuromuscular coordination but not muscle strength-endurance. The reverse results from strength training. More studies are required to confirm effects on other physiological functions, as well as the translation of formal training to functional tasks where current evidence is conflicting. It is desirable that future studies follow similar training and measurement protocols to enhance the quality of data and permit future meta-analyses to strengthen evidence of specificity of effect of different training modes. An area in urgent need of further research is that of exercise dosage. There was little consistency across studies regarding the amount of training. The optimal dose of DCF training to achieve changes in the various physiological functions is unknown. It is also unknown if a certain dosage will affect all physiological functions similarly, or different exercise dosages are required for different physiological effects. Finally, research is urgently required to investigate if exercise effects last into the long term or if a maintenance regime is needed to maintain effects, which is a crucial issue when the aim is to reduce recurrent episodes of neck pain.

### Clinical implications

The findings of the review suggests that there is specificity of response to DCF training, in relation to training cervical neuromuscular coordination and functional sitting postures. These findings were reported in studies with non-specific, non-specified or mix of non-traumatic and whiplash groups. This indicates that DCF training has these specific effects on various neck pain conditions. Deep cervical flexion training seems to have no or small effects on flexor muscle strength, but more research is needed for a definite conclusion. Some physiological functions appear to improve independent of exercise mode such as contraction accuracy. There is conflicting and insufficient evidence for translation of effects of either DCF or strength training to improve performance in light functional tasks. Overall, the findings from this review suggest a single training mode will not address all impaired physiological functions and a rehabilitation program should incorporate multiple training modes specific to the assessed impaired physiological functions.

Of interest 10 of the 12 studies measured the effect of the various training modes on self-reported measures of neck pain and disability (exceptions [33, 36]). While the exercise interventions had different physiological and functional effects, the different training modes reduced self-reported pain and disability in a similar way. Neck pain and disability is commonly the primary, patient-centred outcomes in RCTs. Thus when relief of neck pain is the primary outcome, it would seem not to matter which exercise is prescribed. However this review suggests that this is not the case when the aim is to address impaired physiological functions where specific impairments are best addressed by specific training regimes.

## Conclusion

The findings of this review indicate a consistent trend that DCF training can successfully address impaired neuromuscular coordination within the neck flexor muscles, but cannot address impaired cervical flexor muscle strength and endurance at higher contraction intensities. The small number of studies investigating other physiological functions impacts on the interpretation and strength of the evidence. We can however suggest that a multimodal training regime is required when the aim of management of the patient with a neck pain disorder is to address the impaired physiological function associated with neck pain. Areas for further research are suggested to strengthen knowledge about specificity of effect of modes of training.

## Abbreviations

ARV: Average rectified value
AS: Anterior scalenes
CCFT: Craniocervical flexion test
DCF: Deep cervical flexor
EMG: Electromyography
JPS: Joint position sense
MSF: Mean spectral frequency
MVC: Maximum voluntary contraction
MVIC: Maximum voluntary isometric contraction
ROM: Range of motion
SC: Splenius capitis
SCM: Sternocleidomastoideus
